# In vitro examination of the thrombolytic efficacy of tenecteplase and therapeutic ultrasound compared to rt-PA

**DOI:** 10.1186/s12883-019-1404-5

**Published:** 2019-08-02

**Authors:** Tobias Frühwald, Ulrich Gärtner, Nils Stöckmann, Jan-Henning Marxsen, Carolin Gramsch, Florian C. Roessler

**Affiliations:** 1 Department of Neurology, Justus-Liebig-University Gießen, Klinikstraße 33, 35385 Gießen, Germany; 2 Institute of Anatomy and Cell Biology, Justus-Liebig-University Gießen, Aulweg 123, 35392 Gießen, Germany; 3 Oncology Practice Am Marien-Krankenhaus, Parade 5, 23552 Lübeck, Germany; 4 Department of Neuroradiology, Justus-Liebig-University Gießen, Klinikstraße 33, 35385 Gießen, Germany; 5 Klinik und Poliklinik für Neurologie, Universitätsklinikum Standort Gießen, Klinikstraße 33, 35385 Gießen, Germany

**Keywords:** Stroke, Thrombolysis, Tenecteplase, Ultrasound, Transmission electron microscopy

## Abstract

**Background:**

Optimizing thrombolytic therapy is vital for improving stroke outcomes. We aimed to develop standardized thrombolysis conditions to evaluate the efficacy of tenecteplase (TNK) compared to the current gold standard rt-PA (alteplase), with and without additional ultrasound treatment. We also wanted to introduce a new analytical approach to quantify fibrin fiber density in transmission electron microscopy (TEM).

**Methods:**

In vitro clots that are similar to ex vivo clots concerning their histological condition and their durability were generated from whole blood. For five treatment groups we compared relative clot weight loss (each *n* = 60) and fibrin fiber density in TEM (each *n* = 5). The control group (A) was treated only with plasma. Two groups were designated for each rt-PA (B + C) and TNK (D + E). Groups C and E were additionally treated with ultrasound. Dosages were 50 μg/ml for rt-PA and 30 μg/ml for TNK. Results were evaluated by using analyses of variance (ANOVA) and post-hoc t-tests.

**Results:**

Weight loss was increased significantly for all groups compared to the control group. Both TNK groups showed significantly increased weight loss compared to their counterpart rt-PA group (*p* ≤ 0.001). For TEM only group D showed significantly decreased fibrin fiber density (*p* < 0.05) compared to both rt-PA groups. Ultrasound did not significantly increase dissolution of clots with either method (best *p* = 0.16).

**Conclusions:**

Tenecteplase dissolved clots more effectively than rt-PA with and without ultrasound. A higher sample size could provide more convincing results for TEM.

## Background

Stroke remains one of the leading causes of death and serious long time disability in the world. Additionally, projections estimate a 20% increase in prevalence in adults by 2030 in comparison to 2012 [1]. Therefore, stroke is a clinically and epidemiologically highly relevant and impactful disease, and will remain so in the near future.

About 87% of all strokes are ischemic in nature [1] and optimization of interventional or medicinal recanalization techniques is still substantial to provide better therapeutic options for a large number of patients.

While mechanical thrombectomy promises good results, it is only suitable for proximal vessel occlusions [2]. Therefore, thrombectomy is an addition and not a replacement to intravenous thrombolytic therapy. Concerning thrombolytic drugs, rt-PA (Actilyse®) remains the standard treatment within 4.5 h of stroke onset, regardless of severity. Rt-PA however, offers significant drawbacks, such as the risk of intracranial hemorrhages following treatment and a still limited time window for treatment post onset of symptoms [3] even if this time window is likely to open further [4]. However, up to now, even with rising treatment numbers only 3.5% of all stroke patients are eligible for treatment [5]. Even with treatment, morbidity rates remain at around 50% with mean mortality rates at about 12% [5].

Therefore, it is crucial to find new and improved methods of treatment providing greater efficacy as well as not increasing the intensity and prevalence of side effects.

Alternative fibrinolytic drugs to rt-PA may provide these benefits.

Tenecteplase (TNK, Metalyse®) is one of them. Current guidelines suggest TNK as alternative to alteplase in patients with minor neurological impairment and no major intracranial occlusion [6]. In addition, studies proved TNK at least to be equally effective in large vessel occlusions [7].

Mutations in three parts of the original alteplase molecule raise TNKs specificity to fibrin by a factor of 14–15 and its resistance towards plasminogen activator inhibitor-1 (PAI-1) by a factor of 80 [8, 9]. Furthermore, TNK has a longer half-life of about 22 min [8, 9] compared to a short half-life of 4–9 min of rt-PA [10]. This should lead to a higher affinity to fibrin-rich clots and a more potent and faster clot lysis. Because of the extended half-life, TNK can be given as a single i.v. bolus [9]. This fast and simple route of administration might shorten door-to-needle times and possibly increase thrombolysis rates. Finally, due to less consumption of fibrinogen, plasminogen, and α_2_-antiplasmin TNK has a low systemic fibrinolytic effect and therefore less risk for bleeding [9].

For stroke patients the first randomized controlled trial (RCT) that demonstrated superiority of TNK over rt-PA in terms of clinical efficacy was published in 2012. The effect of 0.1 and 0.25 mg/kg TNK was compared to 0.9 mg/kg rt-PA (only *n* = 25 for each group) less than 6 h after symptom onset. TNK was associated with a significantly better reperfusion and clinical outcome. Stroke patients were selected because of CT perfusion imaging. There was no significant difference in the incidence of intracranial hemorrhages [11]. The validity of these results is limited due to a small sample size, inclusion criteria based on perfusion imaging, and exclusion of patients with mild stroke.

Previously, another double RCT [12] was forced to halt prematurely, because TNK 0.4 mg/kg showed inferiority concerning improvement of NIHSS within 24 h compared to TNK 0.1 and 0.25 mg/kg and a significantly increased frequency of symptomatic intracerebral hemorrhage (sICH). There were no differences in functional outcome at 3 months between either of the TNK groups compared to the rt-PA group.

ATTEST (Alteplase-Tenecteplase Trial Evaluation for Stroke Thrombolysis) [13] found no difference in the percentage of salvaged penumbra at 24–48 h after thrombolysis between TNK 0.25 mg/kg and rt-PA 0.9 mg/kg. Secondary outcomes including clinical improvement at 24 h and functional outcome after 3 months as well as increased risk of sICH or serious adverse events showed also similar results.

Up to now, the NOR-TEST study is the largest (*n* = 1100) RCT [14]. This multicenter trial compared TNK 0.4 mg/kg to rt-PA 0.9 mg/kg in stroke patients within 4.5 h of onset, using only CT for imaging selection. No difference was found with respect to either safety or frequency of gained excellent functional outcome defined as mRS ≤ 1 at 3 months. Interpretation is difficult due to the predominance of mild strokes (median NIHSS at baseline was 4) and high proportions of TIAs and stroke mimics.

The most recently published EXTEND-IA TNK trial showed that given before thrombectomy TNK 0.25 mg/kg was associated with a significantly increased incidence of reperfusion and a better 90-day functional outcome than rt-PA 0.9 mg/kg. The incidence of recovery to independent function did not differ significantly as well as the incidence of symptomatic intracerebral hemorrhage [7]. This study lacks an adequate amount of cases (*n* = 202).

There are many reasons for these inhomogeneous results. Of particular importance are varied TNK doses, various approaches to imaging selection, and different endpoints and case numbers resulting in inconsistent statistical analyses. Coutts et al. provide an overview of the current study situation [15].

A possible addition to any fibrinolytic drug could be ultrasound. As already known, vessels recanalization is facilitated by local application of diagnostic ultrasound when it is applied concurrently with systemic thrombolytic drugs [16–18]. Depending on energy and frequency, ultrasound leads to mechanical fragmentation and cavitation. Both mechanisms increase the penetration and binding affinity of thrombolytic drugs due to reversible disaggregation of fibrin fibers [19]. Systemic adverse reactions such as bleeding are not to be feared due to the local application of ultrasound. Symptomatic intracerebral hemorrhage can be avoided by adjusting the ultrasound parameters to diagnostic settings [17, 20]. However, the occluded vessel has to be depicted using ultrasound imaging to ensure sufficient application of the administered ultrasound energy.

Our aim was to conduct in vitro experiments using a standardized thrombolysis protocol for a reproducible comparison of the thrombolytic efficacy of tenecteplase and rt-PA in either case at their maximum capacity. Furthermore, we investigated the additional effect of therapeutic ultrasound. Finally, in addition to the already established weight loss measurements we introduce a new analytical approach to quantify fibrin fiber density in transmission electron microscopy (TEM).

## Methods

### Clot preparation

All clots were prepared using blood of healthy, human donors, who gave informed consent. After approval of the Ethics Committee of Justus-Liebig-Universtität Gießen (reference number: 206/14) sampling was randomly performed by the local department of transfusion medicine.

Clots were generated using the clot formation protocol developed by Roessler et al. [21]. This protocol ensured a reliable and reproducible preparation of clots staying stable under physiological flow conditions while still retaining adequate lysis rates in experiments. Additionally, clots generated by this protocol showed very similar histological properties to ex vivo clots obtained by thrombectomy [21].

Blood from individual participants were drawn into citrate tubes and centrifuged at two different speeds: 180 *g* for 10 min (Benchtop centrifuge, Allegra 64R, Beckmann-Coulter, Krefeld, Germany) to produce platelet rich plasma (PRP) and 2570 *g* for 10 min (EBA 8S, Hettich, Tuttlingen, Germany) to generate platelet free plasma (PFP).

The resulting supernatants containing PRP and PFP respectively were removed via aspiration. Compensating for individual differences in blood consistency and coagulation quality, plasma from donors of the same blood group was pooled before proceeding. Then, 2.0 ml of PFP and 1.5 ml of PRP were mixed in plastic tubes (REF 55.468.001, Sarstedt) with the addition of 0.5 ml of boundary layer found in PRP tubes between the supernatant and erythrocyte layer. Initiating the clotting process, 0.64 ml of 0.1 mol/l CaCl_2_-solution were added (final concentration: 13.8 mmol/l), and the resulting mixture was incubated in 37 °C for one hour. Afterwards, the resulting clots were placed in new plastic tubes with a blood substitute (Ringer solution, Berlin Chemie Menarini, Berlin, Germany) at 37 °C overnight to ensure full clot retraction and avoid autolytic dissolution that can be observed in clots stored in plasma. At last, the clots were cut to a weight of 300 +**/−** 100 mg, in order to generate more comparable lysis rates.

### Static model

We used a static model our workgroup previously described in other publications for reproducible clot dissolution [22, 23]. For experiments, the model was submerged in 37 °C degassed water. A pump circulating the water prevented heat accumulation. The model we used consisted of a custom-built holding device made out of polyoxymethylene capable of holding up to five Eppendorf pipettes (Safe Seal Gefäß 1,5 ml, REF 72.706, Sarstedt) simultaneously.

The two lateral slots were separated from the others by baffles, allowing for the application of ultrasound to one specific pipette, while shielding the others from unwanted dispersion. To prevent reflection, sound absorbing foamed plastic (SH002, aixFOAM, Eschweiler, Germany), was placed beneath the holders.

### Dose finding experiments

The dose finding was conducted similar to in vitro experiments performed by Holland et al. [24]. The weight loss of clots treated with pooled buffered plasma (pH = 7.4) and TNK or rt-PA respectively (each *n* = 10) was determined by using an analytical balance (Mettler AB104-S Analytical Balance, Mettler-Toledo, Columbus, USA) after 1 h and 2 h for concentrations varying between 0 and 100 μg/ml for rt-PA and between 0 and 40 μg/ml IU for TNK.

### Quantitative weight loss experiments

The same analytical balance was used to determine the percentage of mass loss in an one hour experiment. The experiment was conducted in 5 groups (each *n* = 60). In the control group A clots were treated without a fibrinolytic agent, but solely with buffered plasma (pH = 7.4).

Groups B and C were treated with rt-PA at a dosage of 50 μg/ml and additional ultrasound in group C. For the duration of the experiment, each clot was placed into a new container with fresh rt-PA and plasma every 15 min. This renewal procedure accounted for the short half-life of rt-PA and simulated a clinical situation with continuous rt-PA-application.

Groups D and E were treated with TNK at a dosage of 30 μg/ml. As TNK has a longer half-life and can therefore be applied as a single bolus injection, the renewal process from groups B and C was only simulated by lifting the clots out of their container and then placing them back into it every 15 min during the experiment. The same procedure was performed with clots of the control group A. This ensured that every clot undergoing a lysis procedure was exposed to the same mechanical effects that might influence the results of the weight loss experiment.

### Exposure to therapeutic ultrasound (sonothrombolysis, STL)

In addition to the treatment with their respective fibrinolytic agents, groups C and E were exposed to pulsed wave ultrasound (US) (Sonos2500, Hewlett-Packard, Andover, MA, USA) for one hour operating at 2.0 to 2.5 MHz. We used TCCS (**t**ranscranial **c**olor-**c**oded **m**ode) with an anti-aliasing threshold of 0.25 m/s and a power threshold of 100%. Spatial-peak temporal-average intensity (*I*_SPTA_) was 0,179 W/cm^2^. The mechanical index was 0.6. This system is equivalent to commonly used transcranial diagnostic imaging systems in a clinical setting. Clots and transducer were immersed in degassed water at 37 °C fixed to each other at a distance of 50 mm, with the clot centered within the focal volume of the ultrasound beam to ensure maximum efficacy.

### Electron microscopy

For electron microscopy, clots were fixed in 1.5% glutaraldehyde and 1.5% formaldehyde in 0.15 M HEPES. Clots were transferred into 20% sucrose solution to avoid squeezing artifacts during further processing. After complete immersion, they were subsequently frozen at − 26 °C. Then, they were broken into small pieces and processed. For these experiments, clots were prepared without boundary layer to prevent that red blood cells conceal the fibrin mesh. For transmission electron microscopy (TEM), samples were postfixed in 1% osmium tetroxide in aqua bidest., stained in half-saturated water uranyl acetate (Merck Darmstadt Germany), dehydrated in an ascending ethanol series and finally embedded in agar 100 resin (Agar scientific Ltd. UK). Five clots from each treatment group (A-E) were processed. For each clot, five ultrathin slices were cut using an ultramicrotome (Reichert Ultracut E ultramicrotome, Leica Microsystems, Wetzlar, Germany) and examined in a transmission electron microscope (Zeiss EM 902, Carl Zeiss AG, Oberkochen Germany). Corresponding to histological evaluation out of these slices one representative cutout with a defined size (5x5μm) was chosen and captured with a slow-scan digital 2 K CCD camera (TRS, Tröndle, Moorenweis, Germany). Then, the contrast of all chosen cutouts was adapted to each other by choosing the same setting of tone histogram (mean value: 1.0; tonal range: 0–255) using a commercial image processing software (Adobe Photoshop CS6 Extended). Finally, these color-corrected cutouts were uploaded by an open source software (ImageJ, Java V.1.8.0_77). Its feature *IMAGE / ADJUST / THRESHOLD* performs an automatic marking of the fibrin fibers and calculates the relation of the marked area to the total image size.

### Calculations and statistics

We performed analyses of variance (ANOVA) and post-hoc t-tests to evaluate statistical differences in quantitative weight loss and fibrin fiber density between treatment groups A-E, in order to determine differences for the factors TREATMENT (with or without thrombolysis), MEDICATION (rt-PA or TNK) and ULTRASOUND (none or application of ultrasound).

Calculations and statistics were performed using IBM SPSS statistics (Version 21.0.0.0, IBM, Armonk, New York, U.S.A.)

## Results

### Dose finding experiments

The weight loss without any thrombolytic treatment was 15.3% after 1 h and 27.7% after 2 h of treatment.

**Fig. 1:**
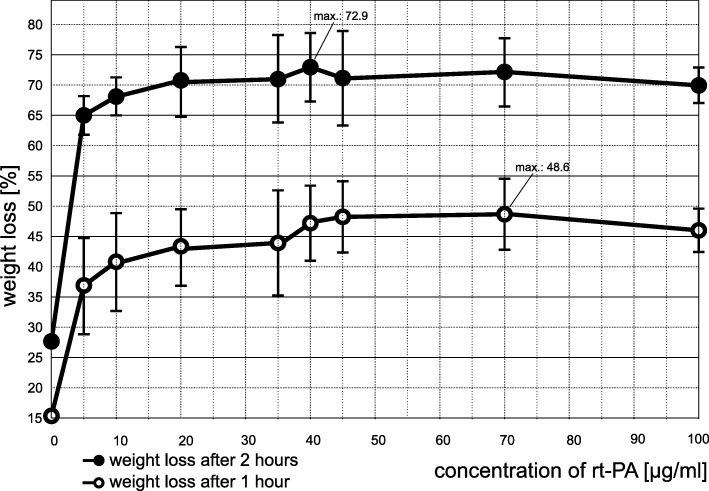
Results from dose finding experiments with rt-PA: Weight loss measurements after 1 and 2 h of treatment with escalating dosages from 0 to 100 μg/ml rt-PA (*n* = 10 for every dosage) were performed to determine the maximum effective dosage for rt-PA. Bars reflect the standard deviation (SD) from the mean. For reasons of clarity, we omitted the SD of the control group (SD for 1 h: 5.7%; SD for 2 h: 7.4%)

For rt-PA we observed a plateau of weight loss between 40 and 50 μg/ml (Fig. 1). Maximum weight loss was 48.6% after 1 h at 70 μg/ml and 72.9% after 2 h at a concentration of 40 μg/ml. In accordance with the observed plateau, we used a rt-PA dosage of 50 μg/ml for all following experiments.

**Fig. 2:**
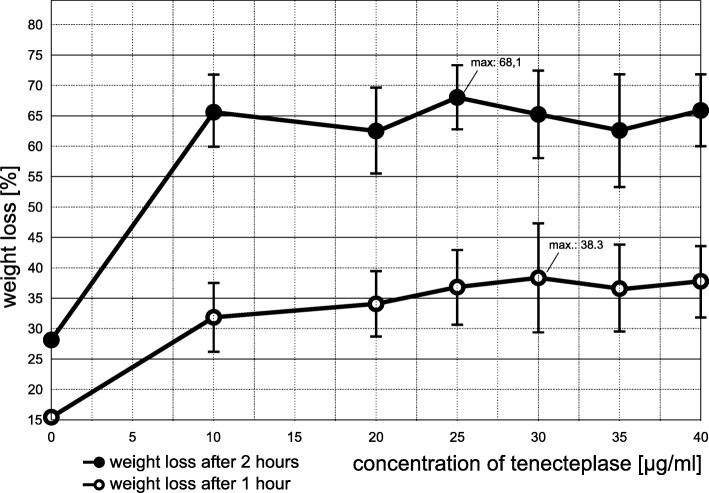
Results from dose finding experiments with tenecteplase (TNK): Weight loss measurements after 1 and 2 h of treatment with escalating dosages from 0 to 40 μg/ml TNK (n = 10 for every dosage) were performed to determine the maximum effective dosage for TNK. Bars reflect the standard deviation (SD) from the mean. For reasons of clarity, we omitted the SD of the control group (SD for 1 h: 5.7%; SD for 2 h: 7.4%)

For TNK maximum weight loss was 38.3% after 1 h at a dosage of 30 μg/ml and 68.1% after 2 h at a dosage of 25 μg/ml (Fig. 2). We used a TNK dosage of 30 μg/ml for all following experiments.

### Quantitative weight loss experiments

**Fig. 3:**
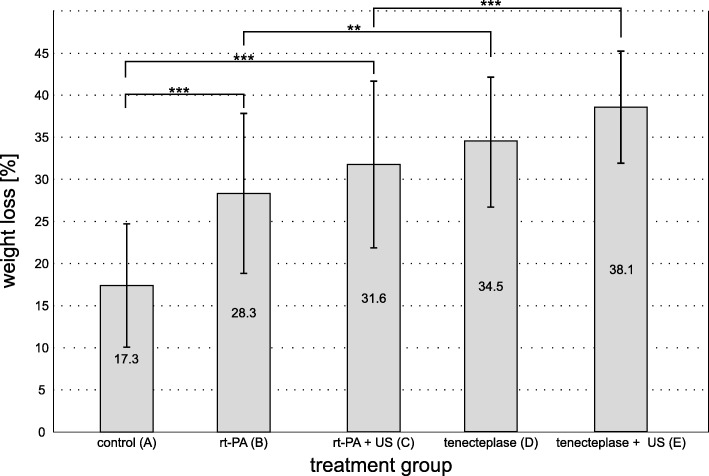
Results from quantitative weight loss experiments. Clot weight loss after 1 h in 5 different treatment groups (each *n* = 60). Group A acted as the control (clots treated only with buffered plasma). Groups B and C were treated with rt-PA (50 μg/ml), groups D and E with TNK (30 μg/ml). Groups C and E additionally received ultrasound application (2 MHz, 179 W/cm^2^). Values are depicted as mean relative weight loss with standard deviation from the mean. Values can be looked up in Table 1. *** *p* < 0.001, ** *p* = 0.001

The measured values are listed in Table 1 and depicted in Fig. 3. Compared to control group A with a weight loss of 17.3 ± 7.4% all other groups showed a significantly increased weight loss (each *p* < 0.001).

**Table 1:** Values of lysis experiments as depicted in Fig. 3 and TEM experiments as depicted in Fig. 4

	A control	B rt-PA	C rt-PA + US	D TNK	E TNK + US
weight loss ± SD [%]	17.3 ± 7.4	28.3 ± 9.6	31.6 ± 10	34.5 ± 8	38.1 ± 6.7
area covered by fibrin ± SD [%]	58 ± 5.2	54.7 ± 3.6	52.9 ± 6.5	42.3 ± 6	45.1 ± 2.3

Results for measured relative clot weight loss of different treatment groups (A-E) as well as for relative area covered by fibrin in TEM for the different treatment strategies are combined. SD: standard deviation from the mean. TNK: tenecteplase; US: color-coded sonography (2 MHz, 0.179 W/cm^2^)

Additionally, both TNK groups D and E showed significantly increased weight losses in comparison to their respective rt-PA counterparts group B and C. Weight loss in group B was 28.3 ± 9.6% compared to group D with 34.5 ± 8% (*p* = 0.001). Weight loss in group C was 31.6 ± 10% compared to group E with 38.1 ± 6.7% (*p* < 0.001).

Both groups with additional STL(C and E) had higher mean weight losses than their corresponding groups without STL (B and D). But this effect did not meet statistical significance. Group C had 3.3% more weight loss than group B (*p* = 0.23). Group E had 3.6% more weight loss than group D (*p* = 0.16).

### Transmission electron microscopy

**Fig. 4:**
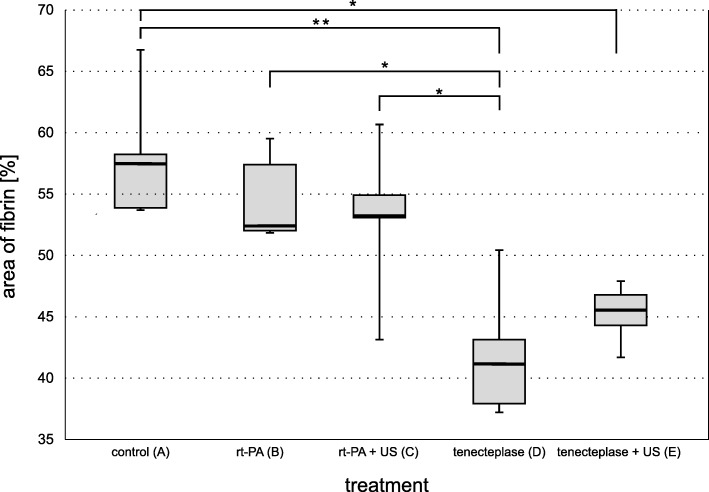
Boxplot for percentage of area covered by fibrin. Semi quantitative analysis was performed on the 5 treatment groups (each *n* = 5) and depicted with representative TEM cutouts**.** The duration of the treatment was 1 h for each treatment. Values can be looked up in Table 1.* *p* < 0.05, ** *p* = 0.001

**Fig. 5:**
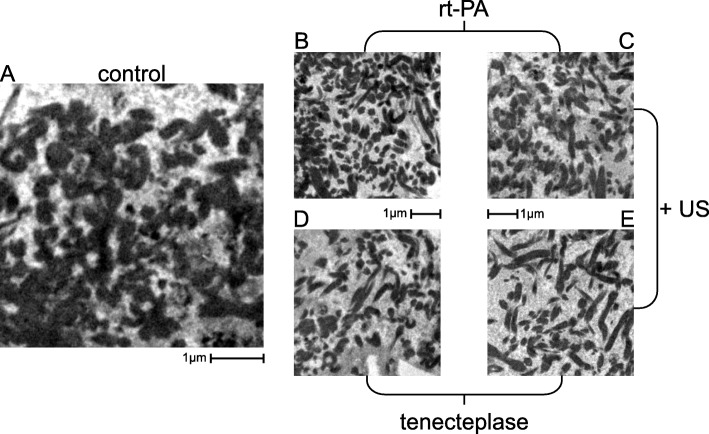
TEM cutouts. Examples of TEM cutouts used for semi quantitative analysis. Treatment group: **a** control group (solely buffered plasma (pH = 7.4). **b** rt-PA (50 μg/ml in buffered plasma). **c** rt-PA (50 μg/ml in buffered plasma) with additional US. **d** TNK (30 μg/ml in buffered plasma). **e** TNK (30 μg/ml in buffered plasma) with additional US. US: 2.0 to 2.5 MHz transcranial color-coded ultrasound (spatial-peak temporal-average intensity: 0,179 W/cm^2^; mechanical index = 0.6)

For TEM the threshold value for the percentage of area covered by fibrin fibers ranged from a maximum of 58% for the control group to a minimum of 42.3% for TNK alone (TNK with STL: 45.1%) (Fig. 4). Values for rt-PA and rt-PA with STL were 54.7 and 52.9%, respectively. All weight loss and fibrin fiber density values were collected in Table 1. Figure 5 depicts representative cutouts. While these results show a trend fitting to the results of the quantitative weight loss experiments, statistical significance for differences from the control group was only proved for both TNK groups (*p* = 0.001 for TNK and *p* = 0.006 for TNK with STL). Group D (TNK) exhibited the lowest density in fibrin fibers, showing an additional significant difference to groups B (*p* = 0.008) and C (*p* = 0.03).

## Discussion

### Dose finding experiments

Our experiments were designed to combine maximum effects of the two different thrombolytics and the influence of additional ultrasound application. Therefore, results cannot be transferred directly to clinical situation.

We used rt-PA at a dosage of 50 μg/ml. This corresponds to about 3.6 mg/kg bodyweight assuming a blood volume of 5000 ml as calculated by the Nadlers formula for a healthy male adult with a body weight of 70 kg and a height of 180 cm. In daily clinical practice, rt-PA is used with a dosage of 0.9 mg/kg bodyweight. TNK was used at a dosage of 30 μg/ml corresponding to approximately 2.1 mg/kg bodyweight. Studies suggest a maximum suitable TNK dosage of 0.4 mg/kg bodyweight [8, 14, 25]. Therefore, the administered dosages in our in vitro experiments exceed therapeutic dosages with a factor of 4 for rt-PA and of 5.25 for TNK.

It has to be mentioned that the translation of the used in vitro drug concentration to drug dosages given in clinical settings by the Nadlers formula is only a rough estimation that disregards all pharmacological and pharmacokinetic differences between the in vitro and the in vivo set up. The validity of our study cannot be derived from this.

There are hardly any references about in vivo concentrations for rt-PA or TNK in blood after their application in clinical settings. We found one study performed on patients with acute myocardial infarction, showing rt-PA concentration to be at about 3 μg/ml at the steady state after intravenous administration [26]. However, in this study, different dosages of rt-PA were used and sample size was only 4. These results can hardly be transferred to clinical settings of patients with acute ischemic strokes. It is a widespread approach to compare the effect of different drugs in vitro in the range of their saturation point to avoid concentration-dependent effects and underestimation. That is what we did in our study. For rt-PA, we found the same saturation value as Holland et al. [24].

Animal experiments and clinical studies are needed to determine a suitable dosage for both thrombolytics regarding safety and efficacy.

### Quantitative weight loss experiments

All treatment groups showed a significantly increased weight loss in comparison to the control group.

In our in vitro experiments, TNK is significantly more effective than rt-PA even if both drugs are used with an additional application of ultrasound. Appropriately, first clinical studies point out that TNK is associated with significantly better reperfusion and clinical outcomes than rt-PA in patients with acute ischemic stroke [7, 11]. This might result from TNKs higher specificity to fibrin.

A statistically significant effect of ultrasound cannot be demonstrated, even though both US groups exhibited more weight loss than their counterpart without US did.

This finding is in line with other in vitro studies and reflects the heterogeneous picture drawn by recent clinical trials and meta-analyses. Several in vitro studies found significantly increased lysis rates after the addition of ultrasound to rt-PA treatment [24, 27], whereas other in vitro studies showed no significant effect [21, 22, 28]. Clinical studies revealed significantly increased recanalization rates, but failed in proving significantly increased recovery from stroke [16, 17]. Only a subgroup analysis of the CLOTBUST trial (patients with pre-treatment NIHSS scores ≥10 points and proximal intracranial occlusions) showed a significantly increased number of sustained complete recanalization and of functional independence at 90 days in the sonothrombolysis group [29]. The Cochrane Stroke Group identified five eligible studies with a total of 223 patients and found that failure to recanalize was lower without clear hazard in the sonothrombolysis group and patients treated with ultrasound and rt-PA were less likely to be dead or disabled at three months [30]. According to a meta-analysis of 10 phase II trials, patients with visible intracranial occlusion treated with sonothrombolysis (*n* = 345) have more than two-fold higher likelihood of achieving both complete recanalization at 2 h and favorable functional outcome (mRS ≤ 2) at three months compared to patients not receiving sonothrombolysis (*n* = 275) [31]. The major limitations of the pooled trials are small sample sizes and no double blindness in randomization and clinical follow-up. The CLOTBUST-ER trial, a double-blind, multicenter, phase III, randomized controlled trial that aimed to investigate efficacy and safety of sonothrombolysis (2 h of 2 MHz pulsed-wave ultrasound) vs rt-PA alone in patients with acute ischemic stroke with NIHSS-scores of 10 or higher, showed that sonothrombolysis is safe but does not improve functional outcome at 90 days compared to rt-PA alone [32]. Results might be affected by three main factors: First, documentation of proximal intracranial occlusion was not required. Second, the operator-independent ultrasound device might have provided less efficient ultrasound exposure. Third, the trail was stopped early because of futility leading to incomplete data concerning functional outcome at 90 days. Accordingly, a new sonothrombolysis trial (TRUST; NCT03519737) has been initiated. This trial investigates recanalization rates before thrombectomy on patients with large-vessel occlusions being transferred from primary to secondary stroke centres randomized to either ultrasound or no ultrasound using an optimized device.

Papadopoulos et al. performed an in vitro experiment that is very similar to ours [33]. In accordance with our results thrombolysis increased with increasing concentration of TNK, which was more effective than thrombolysis in untreated clots or in clots solely treated with ultrasound. The additional application of ultrasound encouraged the thrombolytic efficacy of TNK. This finding substantiates the assumption that ultrasound increases the penetration and binding affinity of thrombolytic drugs due to reversible disaggregation of fibrin fibers by mechanical fragmentation. Ultrasound alone has no meaningful thrombolytic effect. In contrast to our results, in the experiments of Papadopoulos et al. clots treated with TNK and additional ultrasound exhibited a significant greater thrombolysis degree of 25% compared with clots treated with TNK alone. This could be explained by the following differences of the experimental approach:

1. Papadopoulos et al. generated clots by natural coagulation of fresh porcine blood. These clots are very soft and less resistant to thrombolysis especially under flow conditions [34]. Thus, spontaneous lysis of the whole blood clots was about 30% after 30 min. This basically corresponds to the lysis rate of our PRP-clots after two hours. Due to our own experiences, whole blood clots are not suitable for thrombolysis experiments [21].
2. Papadopoulos et al. used a totally different ultrasound setting. The middle frequency of their ultrasound probe was 1 MHz. The bandwidth of our phased-array ultrasound transducer was 2.0. to 2.5 MHz. We used a spatial-peak temporal-average intensity (*I*_SPTA_) of 0,179 W/cm^2^ and a mechanical index of 0.6. Papadopoulos et al. indicate an acoustic power of 20 W sent to the transducer’s surface. It is important to note, that the upper approved limit for diagnostic purposes is 0.72 W/cm^2^. This suggests a markedly lower energy transmission in our experiments derived from commonly used transcranial diagnostic imaging systems in a clinical setting.

Finally, a comparison of the numerical values of the gained results is hampered by the fact, that there are many other differences concerning the experimental setup and the number of conducted experiments for each group (Papadopoulos et al.: *n* = 5; present investigation: *n* = 60).

### Transmission electron microscopy

Corresponding to the quantitative weight loss experiments we demonstrated a loosening of the fibrin network by TEM imaging. Both rt-PA groups did not differ significantly from the control and in contrast to our weight loss experiments fibrin networks showed more loosening for TNK than for TNK combined with ultrasound. Both findings are likely to be due to the small sample size of 5 for each treatment group. We assume that acoustic streaming leads to increased enzymatic fibrinolysis when thrombolytic agents are combined with ultrasound [35, 36]. Evidence suggests that ultrasound reduces clot density by disrupting cross-linked fibrin fibers [37]. This process – although reversible and of short duration – allows fibrinolytic agents to penetrate into the clot more effectively because of an increased contact surface and therefore additional binding sites. The resulting rarefication of the fibrin mesh is irreversible. [37]. This mechanism should be more effective for drugs with an increased specificity to fibrin such as TNK. For a better observation of the rarefication of fibrin meshes, we decided to generate clots without boundary layer to prevent that red blood cells conceal the fibrin mesh. Therefore, we are not able to confirm observations made by Auboire et al. that the main impact of ultrasound is the removal of red blood cells from the clot [38]. Also Petit et al. found that ultrasound alone induces only clot hemolysis without degrading the fibrin network [39]. Presumably, the thrombolytic effect of ultrasound comprises both mechanisms.

Our results confirm that the TEM can be effectively used as an additional method of assessing thrombolysis quality. We are confident that further analyses with larger sample sizes and more optimized selection can enhance the validity of this procedure and provide deeper insights into the mode of action of thrombolysis.

### Limitations of our experimental setup

Investigations of different lysis strategies require a standardized experimental setup. Otherwise, lysis rates of different treatment strategies cannot be compared, which is counterproductive to progress in research and therapy. On the other hand, reproducible in vitro experiments suffer from inevitable limitations in comparison to in vivo therapy. In the case of our model, we must point out the following disadvantages:

1. To prevent further clotting during experimentation we used blood plasma as a blood substitute, nevertheless all blood components that are necessary for performing valid thrombolysis experiments are contained in the experimental setup.
2. Our static model does not factor in the mechanical impact of physiological blood flow on thrombolysis. This however, enables reproducible analysis of enzymatic thrombolysis.
3. In clinical settings of transcranial sonography ultrasound becomes attenuated on its way through the scull and brain tissue. In the present model the attenuation is smaller leading to a higher energy of the insonated ultrasound and therefore to an overestimated thrombolytic effect of the ultrasound. However, we found no statistically significant effect of ultrasound. Therefore, our results may not be strongly falsified.
4. The model cannot consider the complex interaction between clot and endothelium of the sealed vessel nor the mutual influences between coagulation, fibrinolysis, and inflammatory processes. Not much is known about these interactions [40]. Probably vessel occlusion leads to immediate activation of the inflammatory cascade, especially in ischemic areas that are completely occluded and therefore hypoxic, with activation of the complement system, blood platelets and endothelial cells [41]. However, the whole immunological response extends over a time period of weeks [42]. Therefore, simplifications of our model concerning this field are acceptable, because our experiments are centered around acute treatment of stroke.
5. No in vitro clot formation protocol will ever reflect the natural process of in vivo clotting in detail. Our results just apply to PRP-clots. However, previous publications conducted by our workgroup showed that PRP-clots are histologically similar to ex vivo clots gained from patients who underwent thrombectomy [21].
6. The initial mass of the analyzed clot types vary considerably. Throughout our experiments, heavier clots with a greater surface show higher lysis rates probably because of their increased contact surface with the surrounding fluid. Therefore, all data are calculated as relative measures to reduce this source of error.
7. The dosages of thrombolytics we used exceed clinically used dosages by a factor of 4 and 5.25, respectively. As we looked for a plateau of the lysis effect in our dose finding, we are confident that both treatments could unfold their full thrombolytic potential on the one hand without overestimating their efficacy on the other hand. However, our experiments cannot factor in any side effects of treatment, like intracranial hemorrhage.
8. Because of the different half-lives of rt-PA and TNK, the solution of buffered plasma and rt-PA was renewed every 15 min during the lysis experiments. In contrast, the solutions of TNK were kept the same during the whole treatment. Finally, this is also only an approximation.
9. The semi-quantitative analysis of the area of fibrin in the TEM images is based on a small sample number. Furthermore, image selection and data processing can potentially skew results. TEM experiments with a larger sample size would further solidify the validity of this particular method for evaluating the quality of thrombolysis.

Despite all these limitations and the restricted transferability of our results to patient treatment an increased thrombolytic potential of TNK compared to rt-PA can be derived from our experiments.

## Conclusions

In our experimental setup TNK is more effective than rt-PA both with and without ultrasound as measured by relative weight loss of the clots. Measured by fibrin fiber density in TEM imaging efficacy of TNK alone is also higher than that of rt-PA alone or in combination with ultrasound. Our standardized experiments confirm the hypothesis of TNK being a more potent alternative to rt-PA due to its increased specificity to fibrin. In contrast, ultrasound causes neither significantly increased weight loss nor reduction of fibrin fiber density in TEM imaging. Future studies should focus on improved ultrasound settings. TEM imaging provides deeper insights into the mode of action of (sono)thrombolysis.

## Data Availability

The datasets that were used for the present study are available from the corresponding author on reasonable request.

## Abbreviations

CT: Computed tomography
mRS: modified Ranking scale
NIHSS: National Institutes of Health Stroke Scale
PAI-1: Plasminogen activator inhibitor-1
PFP: Platelet free plasma
PRP: Platelet rich plasma
RCT: Randomized controlled trial
rt-PA: Recombinant tissue plasminogen activator (alteplase)
sICH: Symptomatic intracerebral hemorrhage
STL: Sonothrombolysis
TCCS: Transcranial color-coded mode
TEM: Transmission electron microscopy
TIA: Transient ischemic attack
TNK: Tenecteplase
US: Ultrasound
